# The Morphological Stenosis Pattern of the Caroticoclinoid Foramen

**DOI:** 10.3390/diagnostics15010076

**Published:** 2024-12-31

**Authors:** Ioannis Paschopoulos, George Triantafyllou, Panagiotis Papadopoulos-Manolarakis, Sabino Luzzi, Nektaria Karangeli, George Tsakotos, Renato Galzio, Maria Piagkou

**Affiliations:** 1 Department of Anatomy, School of Medicine, Faculty of Health Sciences, National and Kapodistrian University of Athens, Goudi, 11 527 Athens, Greece; johnpascho@gmail.com (I.P.); georgerose406@gmail.com (G.T.); nekkarangeli@gmail.com (N.K.); gtsakotos@gmail.com (G.T.); 2 Department of Neurosurgery, General Hospital of Nikaia-Piraeus, 18 454 Nikaia, Greece; p.papado89@gmail.com; 3 Department of Clinical-Surgical, Diagnostic and Pediatric Sciences, University of Pavia, 27100 Pavia, Italy; sabino.luzzi@unipv.it (S.L.); renato.galzio@gmail.com (R.G.)

**Keywords:** caroticoclinoid bar, caroticoclinoid foramen, internal carotid artery, compression, clinical anatomy, variation

## Abstract

**Background**: The caroticoclinoid bar (CCB) or caroticoclinoid foramen (CCF) represents a well-described ossified variant of the skull base. It corresponds to an osseous bridge (resulting after homonymous ligament ossification) between the anterior and middle clinoid processes (ACPs and MCPs) surrounding the internal carotid artery (ICA)’s cavernous segment. Although extensive research has been performed on this clinically significant entity, only a few studies have been conducted on its effect on the ICA. The current study on dried skulls, using computed tomography (CT) and computed tomography angiography (CTA) scans, aimed to investigate the CCB’s presence and potential morphological stenosis patterns. **Methods**: One hundred (100) dried adult skulls and one hundred sixty (160) skulls from CT scans of patients were obtained (a total of 520 observations). To further calculate the ICA diameter (at the ACP-MCP region) and correlate the resulting dimeters with all potential morphological stenosis patterns of the CCB, thirty (30) CTAs of patients free of the variant were selected. **Results**: Concerning the osseous pattern morphology, of the total of 520 sides, the CCB was identified in 17.1%, the complete variant (creating a caroticoclinoid foramen-CCF) was calculated in 11.5%, and the incomplete one was calculated in 5.6%. No side, sex, or age impact was identified for the CCB presence. Concerning the ICA dimensions, its diameter was calculated to be between 4 and 5 mm. Thus, we observed three morphological stenosis patterns of the CCF. A low-risk pattern of stenosis (>5 mm diameter) was observed in 40 CCFs (44.9%), an intermediate risk of stenosis (4–5 mm diameter) in 38 CCFs (38.2%), and a high risk of stenosis (<4 mm diameter) was depicted in 15 CCFs (16.8%). **Conclusions**: In the present study, we investigated the CCF presence and potential morphological stenosis patterns by calculating and correlating the ICA diameter. In 16.8% of the current sample with CCFs (irrespective of their morphology), we observed that the ICA is probably at a high risk of compression. Radiologists and neurosurgeons intervening in the area should preoperatively diagnose the possibility of ICA compression in this area.

## 1. Introduction

The cerebral arterial circle provides the brain with an arterial supply composed of the internal carotid and vertebrobasilar systems. The internal carotid artery (ICA) emanates from the common carotid artery bifurcation in various vertebral levels, most commonly at the third cervical vertebra (C3) [1]. The ICA is divided into seven segments: the cervical (C1), the petrous (C2), the lacerum (C3), the cavernous (C4), the clinoid (C5), the ophthalmic/supraclinoid (C6), and the communicating/terminal (C7) segments [2]. Various anatomical factors can contribute to ICA stenosis or compression, which could be life-threatening regardless of the reference level. Multiple studies have linked ICA morphological variability with alterations in normal arterial flow to the brain [1,3,4]. Paulsen et al. [3] identified that the ICA had a curved course in 26.2% of the dissected heads, which could lead to a retropharyngeal course and result in clinical complications. In contrast, Triantafyllou et al. [4] observed that when the ICA is elongated, it is closer to the styloid process (SP), potentially increasing the risk of ICA dissection. One such cause, albeit rare, is Eagle syndrome, which necessitates assessing the elongated SP or the ossified stylohyoid ligament [5]. Although the optimal approach to preventing recurrent ICA cervical segment (cervical artery) dissection related to Eagle syndrome remains under debate, conservative management presents a viable option [5]. These studies suggest that the ICA’s morphological variability could disrupt normal arterial flow to the brain.

The skull base exhibits crucial variants for anatomists, interventional radiologists, and neurosurgeons. Various skull variants have been described, including ossified ligaments to a variable degree and accessory foramina [6,7]. In the middle cranial fossa, the sphenoid bone features limited ligaments associated with clinoid processes. The caroticoclinoid ligament (CCL) is found between the anterior and middle clinoid processes (ACPs and MCPs). The anterior interclinoid ligament is between the anterior and posterior clinoid processes (ACPs and PCPs), and the posterior interclinoid ligament is situated between the MCPs and PCPs. Although ligaments are typically dense and made of fibrous connective tissue, they are often variably (partially or entirely) ossified, primarily as a result of the aging process [8].

Sphenoid bone variants can occur frequently, which may lead to intraoperative complications and increase the risk of adverse events. One such variation is CCL (complete or partial) ossification, resulting in a complete or incomplete caroticoclinoid foramen or bar (CCF or CCB) between the ACPs and MCPs. The variable ossification degree can result in a full or partially ossified CCB, defined as greater than 25% ossification. The CCF is an essential structure due to its location near the cavernous sinus, sphenoid sinus, pituitary gland, and the closely related ICA. When present, the CCF encircles the ICA, and in cases of a transverse diameter lower than that of the ICA, morphological changes in the ICA and arterial compression may occur [9]. Nevertheless, due to the complex interplay of neuronal and vascular structures, a detailed understanding of the regional anatomy is crucial for achieving optimal surgical outcomes when operating para-cavernously [10].

Considering the substantial clinical implications of ICA compression or impingement resulting from variably ossified CCBs, the present study using dried skulls and imaging aims to determine the CCF diameter and correlate it with the ICA diameter to explain the potential compression on the ICA after considering all possible morphological ossified patterns of the CCB, highlighting the phenomenon of stenosis.

## 2. Materials and Methods

**Figure 1:**
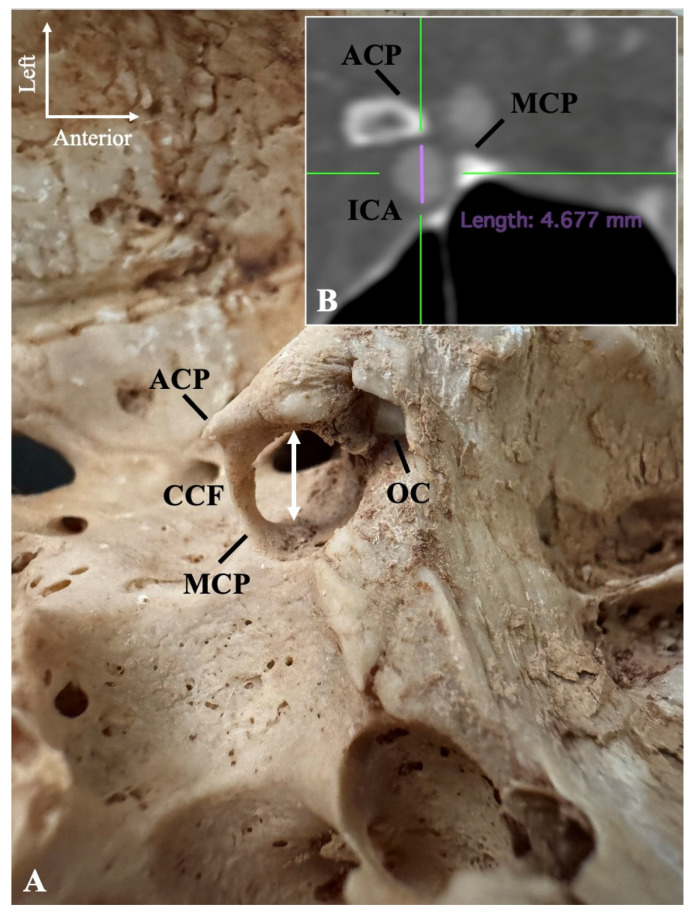
(**A**) The maximum transverse diameter (white arrow) of the caroticoclinoid foramen (CCF); (**B**) the internal carotid artery (ICA) diameter. ACP—anterior clinoid process; MCP—middle clinoid process; OC—optic canal.

The sample included dried skulls, computed tomography scans (CTs), and computed tomography angiographies (CTAs). The osseous (incomplete or complete) bridging between the ACPs and MCPs was considered the CCB formation. The complete CCB was alternatively characterized as the CCF. When present, the CCF transverse diameter was measured (Figure 1A).

One hundred (100) dried adult skulls obtained from the skeletal collection of the Anatomy Department of the Medical School of the National and Kapodistrian University of Athens were investigated for CCB presence and for the calculation of the transverse diameter. The exact ages of the skulls were unknown, and the sex distribution was as follows: 29 were female skulls, 25 were male skulls, and the rest of the skulls were of unknown sex. Moreover, one hundred sixty (160) computed tomography scans (CTs) of the skulls of 85 female and 75 male subjects free of disease were obtained and evaluated. Their mean age was 48.4 years (range from 20 to 79). Thus, a total of 260 skulls were investigated (520 observations).

The investigation was conducted with a 128 multi-slice CT scanner (Philips, Ingenuity CT 128) and documented using the Horos software version 3.3.6 (Horos Project, New York, NY, USA). Evidence was obtained on the multiplanar reconstruction of the axial, coronal, and sagittal slices and their three-dimensional volume reconstruction. To further calculate the ICA diameter (at the ACP-MCP region) and correlate the diameters with all potential morphological stenosis patterns of the CCF, thirty (30) computed tomography angiographies (CTAs) of the brains of 15 male and 15 female patients free of the ossified variant were selected (resulting in a total of 60 observations). None of the chosen patients exhibited any pathology (trauma or any congenital or acquired condition) that could potentially affect the regional anatomy (Figure 1B).

Statistical analysis was performed with IBM SPSS Statistics for MacOS, Version 29 (IBM Corp., Armonk, NY, USA). Nominal data between unpaired observations were compared using the Chi-square test, while McNemar’s test was applied for paired observations. Normality was assessed with the Shapiro–Wilk test. Continuous variables were analyzed based on measurement type: unpaired measurements were evaluated with an independent *t*-test if normality was met; otherwise, the Mann–Whitney U test was used. A paired *t*-test was employed for paired measurements when normality was satisfied. Mean comparisons across more than two groups involved a one-way ANOVA if normal distribution was present; if not, the Kruskal–Wallis test was used. Results are presented as mean and standard deviation unless otherwise specified. A *p*-value less than 0.05 was considered statistically significant.

## 3. Results

**Figure 2:**
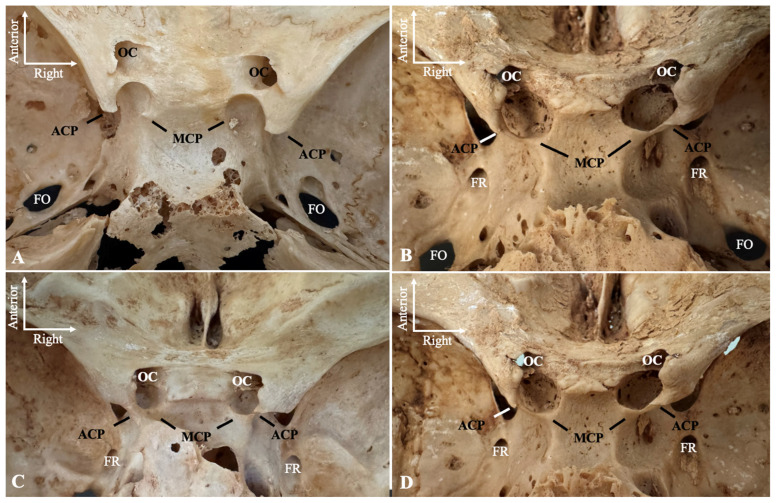
The presence of the caroticoclinoid bar (CCB) in dried skulls. (**A**) A typical skull, (**B**) a bilateral complete caroticoclinoid foramen, (**C**) a complete CCB coexisting with an anterior interclinoid bar and contralateral incomplete CCB, and (**D**) a bilateral complete caroticoclinoid foramen. ACP—anterior clinoid process; MCP—middle clinoid process; OC—optic canal; FO—foramen ovale; FR—foramen rotundum.

**Figure 3:**
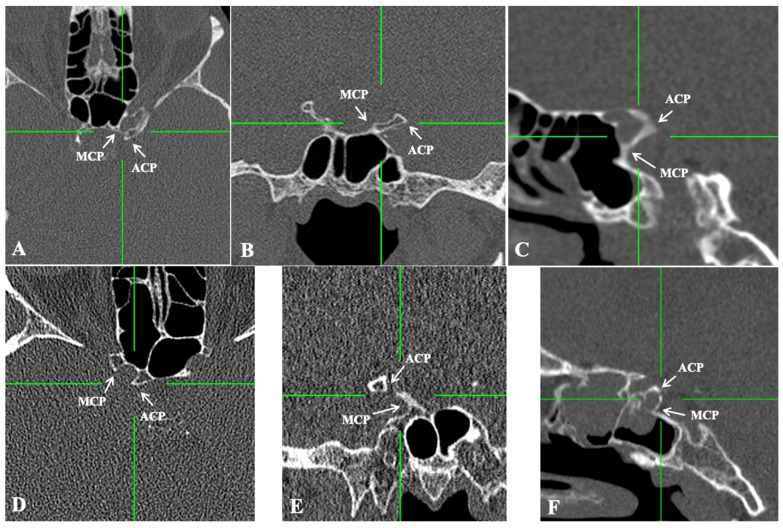
The presence of the caroticoclinoid bar (CCB) in computed tomography scans. (**A**–**C**) Axial, coronal, and sagittal reconstructions of a complete CCB. (**D**–**F**) Axial, coronal, and sagittal reconstructions of an incomplete CCB. ACP—anterior clinoid process; MCP—middle clinoid process.

Of the total of 520 sides, the CCB was identified in 89 sides (89/520, 17.1%), unilaterally in 51 skulls (51/260, 19.6%) and bilaterally in 19 skulls (19/260, 7.3%). The CCB was identified in the complete type in 60 sides (60/520, 11.5%) (Figure 2 and Figure 3) and in the incomplete type in 29 sides (29/520, 5.6%) (Figure 2 and Figure 3). The distributions of the sides and sexes are summarized in Table 1. No differences between the sides, sexes, or ages were identified.

The CCF’s mean maximum transverse diameter was 4.85 mm, ranging between 2.7 and 7.1 mm. The results for the sides and sexes are summarized in Table 2.

The ICA transverse diameter was measured to range between 4.0 mm (minimum) and 5.0 mm (maximum). The mean left ICA diameter was 4.63 ± 0.14 mm, and the mean right ICA diameter was 4.59 ± 0.17 mm with no statistically significant association (*p* = 0.936). The ICA diameter for female patients was 4.48 ± 0.15 mm, and for male patients, it was 4.52 ± 0.33 mm with no statistically significant association (*p* = 0.858).

After correlating the CCF morphometry with the ICA diameter, the ICA potential compression by the CCF was determined and is summarized in Table 3. Based on the minimum ICA diameter (4.0 mm) and the maximum ICA diameter (5.0 mm), three morphological stenosis patterns were recorded. A low risk of compression was considered when the CCF had a diameter of over 5.0 mm. This low-risk type was identified in 40 sides (44.9%). An intermediate risk of compression was observed when the CCF had a diameter between 4.0 and 5.0 mm, which was detected in 34 sides (38.2%). A high risk of compression was observed when the CCF had a diameter of less than 4.0 mm. This high-risk type was identified in 15 sides (16.8%). The compression risk was not associated with the side or the morphology of the CCF (incomplete or complete) type. A female predominance was detected (Table 3).

## 4. Discussion

In the current study, we investigated the CCB’s presence and its morphometry (CCF diameter) while aiming to explore potential ICA compression. We identified that 16.8% of the CCFs had a high risk of arterial compression due to their diameter. This method was not previously performed for the CCF-ICA; however, in an analog modality, it was investigated for suprascapular nerve compression at the suprascapular notch region [11]. The embryological development, morphological variability, and clinical implications of the CCF are further discussed.

### 4.1. Embryological Development

The study conducted by Kawakubo et al. [12] confirmed that the CCB appears at an earlier stage of cranial development. This finding indicates that juvenile population studies may effectively utilize the CCF or CCB as a non-metric cranial trait, which is often used to measure biological distances between different populations [13]. Kier [14] reported an ossified CCF on the right side of a fetus at 30 weeks. In addition to these reports, several authors have noted the CCF’s occurrence in juvenile skulls, including infants aged 21 days, 30 days, 3 months, 7 months, and 15 months [15]; an infant aged 8 months [14]; and a child aged 8 years [16]. Lang [16] proposed that the CCF is a structure surrounded by a cartilaginous preformed bridge during development rather than a product of secondary ossification of ligaments or dural folds. Kier [14] viewed this structure as a developmental anomaly of the embryonic chondrocranium, and Scheuer and Black [17] supported Kier’s perspective. In the study by Kawakubo et al. [12], the CCF was observed in infants and children aged 1.5, 3.6, and 6 years and in fetal crania aged 7 and 10 months.

### 4.2. Caroticoclinoid Bar Morphology

The current study found CCBs in 89 out of 520 cases, resulting in a prevalence of 17.1%. Of these, 60 were categorized as complete CCBs and 29 as incomplete CCBs. Unilateral occurrence was observed in 51 skulls (19.6%), and bilateral occurrence in 19 (7.3%). No significant differences were detected regarding sides, sex, or age. In the study by Natsis et al. [18], a CCB was found in 60.2% of cases. In contrast to the present study, in the study by Natsis et al. [18], partial CCBs appeared more commonly (36.6%) than complete ones (23.6%). The CCF was symmetrical on both sides and genders. Previous research has reported variability in the frequencies of complete and incomplete CCBs. A meta-analysis by Skandalakis et al. [19], which analyzed 26 studies involving 7521 subjects and 14,449 sides, estimated an overall pooled prevalence of 32.6%. When evaluated by study type, CT-based studies revealed a prevalence of 23.1%. In contrast, the present study identified a CCB frequency of 17.1% (89 out of 520), which is lower than the meta-analysis findings. Moreover, the meta-analysis highlighted a significant difference in CCB prevalence between sides, with a notable right-sided predominance. The prevalence of complete and incomplete CCBs varies significantly among ethnic groups; however, our study did not reveal any significant laterality (side asymmetry). Considerable variability in reported frequencies extends across studies, with rates ranging from 9% to 74% depending on the population examined. In the present study, the prevalence of complete CCBs was 11.5%, while incomplete CCBs occurred at a frequency of 5.6% (Table 4).

### 4.3. Caroticoclinoid Foramen Morphometry

Regarding the CCF morphometry in our study, the CCF’s mean transverse diameter was measured at 4.85 mm, ranging from 2.7 mm to 7.1 mm. Given the variability in ICA diameters, which typically range between 4.0 mm and 5.0 mm, the risk of arterial compression varies accordingly. Ozdogmus et al. [10] found the ICA diameter to be 3.73 mm ± 0.71 mm on the right (range 2.28–5.65 mm) and 3.87 mm ± 0.72 mm on the left side (range 1.93–5.50 mm), while Erturk et al. [22] found the ICA’s diameter to be 4.51 ± 0.44 mm (range of 3.8–5.4 mm). Thus, the compression risk is considered low when the CCF diameter exceeds 5.0 mm, moderate when the diameter falls between 4.0 mm and 5.0 mm, and high when the diameter is below 4.0 mm.

In their study, Ozdogmus et al. [10] reported an average CCF diameter of 4.98 ± 0.49 mm on the left and 4.94 ± 0.70 mm on the right side, and these findings closely align with the results of our study. Furthermore, their study recorded the ICA’s external diameter as 3.87 ± 0.72 mm on the left side and 3.73 ± 0.71 mm on the right, which supports our assessment regarding the potential risk of ICA compression. However, statistical evaluations showed no significant correlations between sex, age, side, or incomplete or complete ossification of the CCL. Erturk et al. [22], using comparable measurements in a cadaveric study, reported that the CCF’s mean transverse diameter was 5.32 ± 0.52 mm bilaterally, with respective diameters of 5.33 ± 0.3 mm and 5.32 ± 0.73 mm for the right and left sides. These measurements are marginally higher than those observed in our study and in Ozdogmus’s study. In a similar cadaveric investigation, Kapur et al. [27] determined the CCF’s mean transverse diameter to be 5.32 ± 0.52 mm on the right and 5.21 ± 0.73 mm on the left side. Supporting these findings, Natsis et al. [18] reported the mean transverse diameter of the CCF as 5.56 ± 0.94 mm on the left and 5.32 ± 0.54 mm on the right side. These results underline that the CCB’s diameter can vary significantly depending on the population studied.

### 4.4. Clinical and Surgical Significance

The clinical relevance of CCBs lies in their potential to displace the ICA, which may hinder the effective mobilization of the ICA and limit access to the cavernous sinus, thereby heightening the risk of intraoperative complications—particularly ICA injury—during anterior or middle clinoidectomy [19]. Additionally, CCBs may entrap and compress neurovascular structures, potentially leading to lesions from mechanical irritation or sustained pressure. Specifically, the ICA diameter varies between 4.00 mm and 5.00 mm, making the risk of compression different in each case. Thus, as we suggested, patients with a transverse diameter of the CCF lower than 4.00 mm have a significant risk of compressing the ICA, resulting in a potentially severe blood flow reduction. Furthermore, a foramen that fully develops in infancy may narrow progressively with age due to bone deposition at its margins, which can also exert compressive effects on traversing structures [34]. The CCF is an underestimated structure with important neuronal and vascular relations. It is clinically and surgically crucial as it may result in insufficient blood supply to the brain. Understanding the detailed anatomy of the clinoidal area and the CCF is important before neurosurgical procedures in the sellar or parasellar region.

Anukulsampan et al. [35] described the MCP as an essential structure in skull base anatomy for neurosurgeons. Their study in Thai individuals revealed a relatively high prevalence rate; however, most needed to be completed and prominent. In patients with sellar lesions, the prevalence of MCPs was significantly lower than in cases without sellar pathology. The prevalence of CCF was relatively low compared with previous studies.

Serrano-Rubio et al. [36] emphasized that the microsurgical treatment of paraclinoid aneurysms is a complex undertaking that requires ACP removal to ensure adequate surgical exposure. This procedure poses significant technical challenges due to the ACP’s close association with critical neurovascular structures. Moreover, variations in the parasellar region, such as the CCF, can introduce additional difficulties and heighten the risk of surgical complications. The preoperative identification of ossified structures is essential to detect cases of clinoid processes, determine the extent of ossification and pneumatization, and avoid fatal cerebral complications when aneurysms are present [30].

### 4.5. Limitations

We must acknowledge a few limitations of the current study. The sample was derived from a specific geographic region (Athens, Greece), although the sample was considered adequate for our study (*n* = 260 skulls). The CCBs were classified based on their diameters irrespectively of their type (complete or incomplete) to unify our results.

## 5. Conclusions

Three morphological stenosis patterns were recorded based on the ICA diameter between the ACP and PCP. The most important finding of the present study is that 16.8% of the CCFs pose a high risk of compression to the ICA (a diameter of less than 4 mm). Nevertheless, a female predominance was observed for this type of compression. Future research based on this method will enhance our knowledge about potential differences in sex, side, or nationality. When operating in the area, knowledge of CCB variants is important for radiologists and neurosurgeons.

## Figures and Tables

**Table 1:** The presence of the caroticoclinoid bar (CCB) regarding sides and sexes.

Parameters	Incomplete CCB, *n* (%)	Complete CCB, *n* (%)
Total	29 (5.6)	60 (11.5)
Left	12 (4.6)	32 (12.3)
Right	17 (6.5)	28 (10.8)
*p*-value	0.339	0.583
Female	14 (6.1)	30 (13.2)
Male	7 (3.5)	25 (12.5)
*p*-value	0.161	0.101

**Table 2:** The caroticoclinoid foramen (CCF)’s transverse diameter regarding sides and sexes. The results are presented as the mean (standard deviation).

Parameters	CCB Diameter, Mean (SD)
Total	4.85 (0.93)
Left	4.80 (0.92)
Right	4.90 (0.94)
*p*-value	0.973
Female	4.43 (0.74)
Male	5.21 (1.00)
*p*-value	0.141

**Table 3:** The morphological stenosis pattern of the caroticoclinoid foramen (CCF) is based on its transverse diameter compared to the internal carotid artery (ICA)’s diameter. The Chi-square test was performed (with Bonferroni correction), and * indicates statistically significant results. Note that the sexes of 56 dried skulls in our collection were unknown. The risk is also indicated by color (low risk—green color; intermediate risk—orange color; high risk—red color).

Parameters	Low Risk, *n* (%) (>5.0 mm)	Intermediate Risk, *n* (%) (4.0–5.0 mm)	High Risk, *n* (%) (<4.0 mm)
Total	40 (44.9)	34 (38.2)	15 (16.8)
Left	18 (40.9)	19 (43.1)	7 (15.9)
Right	22 (48.8)	15 (33.3)	8 (17.7)
*p*-value	0.623		
Female	11 (25.0)	20 (45.4)	13 (29.5)
Male	19 (59.3)	11 (34.3)	2 (6.2)
*p*-value	0.002 *		
CCF Incomplete	25 (28)	23 (25.8)	11 (12.3)
CCF Complete	15 (16.8)	11 (12.3)	4 (4.4)
*p*-value	0.769		

**Table 4:** The morphological variability of the caroticoclinoid bar (CCB) in the current published literature.

Study	Year	Number of Skulls	Caroticoclinoid Bar Frequency		
			Complete %	Incomplete %	Total %
Keyes et al. [15]	1935	2187	-	-	34.8
Lee et al. [20]	1997	73	4.1	11.6	15.7
Peker et al. [21]	2002	452	-	-	34.2
Ozdogmus et al. [10]	2003	50	27	18	45
Erturk et al. [22]	2004	171	8.8	14.9	35.7
Gupta et al. [23]	2005	35	8.6	11.4	20
Archana et al. [24]	2010	250	5.2	6.8	12.00
Boyan et al. [25]	2011	34	-	-	35.3
Aggarwal et al. [26]	2012	70	3.0	13.4	16.4
Kapur et al. [27]	2012	200	7.0	9.8%	16.8
Ota et al. [28]	2015	72	-	-	16.6
Suprasanna et al. [29]	2017	44	-	-	22.2
Natsis et al. [18]	2018	123	23.6	36.6	60.16
Efthymiou et al. [30]	2018	76	20.4	46	74
Touska et al. [31]	2019	240	10.0	5	17.5
Zdilla et al. [32]	2019	101	3.5	2.5	18.1
Priya et al. [33]	2021	100	2	7	9
Nikolova et al. [34]	2023	315	9.5	4.8	14.3
Present study	2024	260	-	-	26.9

## Data Availability

All the data are available upon reasonable request from the corresponding author (Professor Maria Piagkou—mapian@med.uoa.gr).
